# Unravelling patient pathways in the context of antibacterial resistance in East Africa

**DOI:** 10.1186/s12879-023-08392-9

**Published:** 2023-06-19

**Authors:** Katherine Keenan, Kathryn J. Fredricks, Mary Abed Al Ahad, Stella Neema, Joseph R. Mwanga, Mike Kesby, Martha F. Mushi, Annette Aduda, Dominique L. Green, Andy G. Lynch, Sarah I. Huque, Blandina T. Mmbaga, Hannah Worthington, Catherine Kansiime, Emmanuel Olamijuwon, Nyanda E. Ntinginya, Olga Loza, Joel Bazira, Antonio Maldonado-Barragán, VAnne Smith, Arun Gonzales Decano, John Mwaniki Njeru, Alison Sandeman, John Stelling, Alison Elliott, David Aanensen, Stephen H. Gillespie, Gibson Kibiki, Wilber Sabiiti, Derek J. Sloan, Benon B. Asiimwe, John Kiiru, Stephen E. Mshana, Matthew T. G. Holden, HATUA Consortium, Benjamin Sunday, Pendo Ndaki, Fernando Benitez-Paez, Madeleine Clarkson, Xuejia Ke, Eveline T. Konje

**Affiliations:** 1 School of Geography and Sustainable Development, University of St. Andrews, St Andrews, KY16 9AL UK; 2 Makerere University, Kampala, Uganda; 3 Catholic University of Health and Allied Sciences, Mwanza, Tanzania; 4 Kenya Medical Research Institute, Nairobi, Kenya; 5 Kilimanjaro Clinical Research Institute and Kilimanjaro Christian Medical University College, Moshi, Tanzania; 6 NIMR-Mbeya Medical Research Centre, Mbeya, Tanzania; 7 Mbarara University, Mbarara, Uganda; 8 Brigham and Women’s Hospital, Boston, USA; 9 London School of Hygiene & Tropical Medicine, London, UK; 10 Medical Research Council/Uganda Virus Research Institute and London School of Hygiene & Tropical Medicine Uganda Research Institute, Entebbe, Uganda; 11 University of Oxford, Oxford, UK; 12 Africa Research Excellence Fund, London, UK

**Keywords:** Antibacterial Resistance, Africa, Antibiotics, Treatment seeking, Healthcare system, Urinary tract Infection, Mixed methods, Patient pathways

## Abstract

**Background:**

A key factor driving the development and maintenance of antibacterial resistance (ABR) is individuals’ use of antibiotics (ABs) to treat illness. To better understand motivations and context for antibiotic use we use the concept of a patient treatment-seeking pathway: a treatment journey encompassing where patients go when they are unwell, what motivates their choices, and how they obtain antibiotics. This paper investigates patterns and determinants of patient treatment-seeking pathways, and how they intersect with AB use in East Africa, a region where ABR-attributable deaths are exceptionally high.

**Methods:**

The Holistic Approach to Unravelling Antibacterial Resistance (HATUA) Consortium collected quantitative data from 6,827 adult outpatients presenting with urinary tract infection (UTI) symptoms in Kenya, Tanzania, and Uganda between February 2019- September 2020, and conducted qualitative in-depth patient interviews with a subset (*n* = 116). We described patterns of treatment-seeking visually using Sankey plots and explored explanations and motivations using mixed-methods. Using Bayesian hierarchical regression modelling, we investigated the associations between socio-demographic, economic, healthcare, and attitudinal factors and three factors related to ABR: self-treatment as a first step, having a multi-step treatment pathway, and consuming ABs.

**Results:**

Although most patients (86%) sought help from medical facilities in the first instance, many (56%) described multi-step, repetitive treatment-seeking pathways, which further increased the likelihood of consuming ABs. Higher socio-economic status patients were more likely to consume ABs and have multi-step pathways. Reasons for choosing providers (e.g., cost, location, time) were conditioned by wider structural factors such as hybrid healthcare systems and AB availability.

**Conclusion:**

There is likely to be a reinforcing cycle between complex, repetitive treatment pathways, AB consumption and ABR. A focus on individual antibiotic use as the key intervention point in this cycle ignores the contextual challenges patients face when treatment seeking, which include inadequate access to diagnostics, perceived inefficient public healthcare and ease of purchasing antibiotics without prescription. Pluralistic healthcare landscapes may promote more complex treatment seeking and therefore inappropriate AB use. We recommend further attention to healthcare system factors, focussing on medical facilities (e.g., accessible diagnostics, patient-doctor interactions, information flows), and community AB access points (e.g., drug sellers).

**Supplementary Information:**

The online version contains supplementary material available at 10.1186/s12879-023-08392-9.

## Background

Antibacterial resistance (ABR) is a significant global health threat which compromises the treatment of infections with antibiotics (ABs). ABR was associated with an estimated 4·95 million deaths globally in 2019 [1]. Such deaths are highest in the Sub-Saharan African region, [1] where there is high burden of infectious diseases and fewer resources to tackle ABR. The process of ABR is influenced by the way individuals use ABs, which is, in turn, impacted by social, political, and economic systems operating on a variety of scales [2–5]. It is therefore crucial to understand treatment-seeking behaviours, or ‘patient pathways’ people take when they are unwell, how these relate to AB use, and the potential presence of ABR [6–8]. This study takes as its central focus individual patient pathways, as told by patients in Kenya, Tanzania, and Uganda, and interrogates these behaviours within their social, economic, and political contexts.

Self-treatment with ABs is one example of ‘inappropriate’ individual treatment-seeking behaviour that is posited to contribute to ABR, [9] but which is influenced by a plethora of social and structural factors. A recent mixed-methods paper from six low- and middle-income countries (LMICs) showed that AB self-treatment was common: reported by 55% of respondents surveyed in Vietnam, 46% in Bangladesh, and 36% in Ghana [10]. Propensity for AB self-medication, while influenced by individual characteristics such as socioeconomic status is affected by structural determinants, including AB dispensing regulations, availability of ABs through alternative providers (e.g., drug sellers), and public trust in different types of healthcare providers [11]. In LMICs, some patients choose to self-treat with ABs rather than access them via prescription because of reduced access and perceived deficiencies in the formal healthcare system, such as long queues, short consultation times, and high out-of-pocket expenses [12, 13]. Other influencing factors include recurrent episodes of infections, cultural beliefs and practices, and symptoms stigma [11].

The structure of healthcare systems is vital for understanding patient pathways. Healthcare and treatment landscapes in LMICs are sometimes described as ‘pluralistic’ [14], which typically means there are multiple sources of public and private clinics, alongside pharmacists, drug sellers, and complementary and traditional/herbal medicine providers. The East African countries studied here are no exception, but with some notable differences between them. Kenya has higher per capita health care expenditure than Tanzania or Uganda, and while out-of-pocket costs are reducing year on year, they still make up a substantial share of spending in all three countries [15]. Health insurance coverage is far from universal [16], but is highest in Kenya, lower in Tanzania [17], and lowest in Uganda, where development of a national health insurance scheme is ongoing [18]. Finally, while drug sellers and pharmacies play a vital role in providing access to medication in all three countries [19], access to ABs without prescription through these outlets is relatively common [10, 18, 20–22].

In this paper, we focus on urinary tract infections (UTIs) as a lens for understanding treatment-seeking and ABR more generally. UTIs are bacterial illnesses that are usually treated with ABs, [23] but which can be stigmatizing and reduce quality of life [24]. Globally, over 150 million individuals are diagnosed with UTIs each year, most of whom are women [25]. Empiric use of ABs to treat UTIs contributes to ABR of the uropathogens responsible (typically *Escherichia coli* and other *Enterobacteriacae)*, and presents a growing global challenge [26]. In East Africa, high community prevalence of UTIs, combined with high levels of AB self-medication, may further exacerbate ABR, [23] particularly considering self-management for UTI symptoms is extremely common [27, 28].

### Research questions

The study aims to assess the socioeconomic, attitudinal, and contextual factors associated with patients’ treatment-seeking pathways for UTI-like symptoms in Kenya, Tanzania and Uganda and explore how key pathway points intersect with AB use. We focus on three aspects theoretically related to the ABR: patients self-treating rather than seeking help at a medical facility, having multi-step pathways (e.g., multiple treatment attempts), and AB consumption during the pathway. We use mixed-methods data to explore the lived experience of treatment-seeking and shed light on situational barriers and facilitators of such behaviours.

## Methods

### Context and theoretical approach

This study is part of a multi-country interdisciplinary consortium “Holistic Approach to Unravel Antibacterial Resistance in East Africa (HATUA)” [29]. We conceptualise ABR as an assemblage of interconnected, multi-scalar social, political, and biological influences (see Fig. 1 in [29]). At the heart of this complexity is the ‘patient pathway’, nested in a pluralistic healthcare landscape comprising various formal and informal healthcare providers [30]. Pathway analysis has been used to investigate care for tuberculosis, [6] abortion, [31] and cancer [32] but has rarely been used to understand AB use [8]. Incorporating medical syncretism, the patient pathway may contain any number of steps, and include delays in seeking treatment, different choice of provider, self-treatment and self-medication, and differences in regimen adherence. Rather than a linear sequence of actions, [7] the patient pathway can be iterative and repetitive. To unravel this complexity, we analyse qualitative and quantitative data about AB use pathways in parallel. We avoid the assumption that human behaviour is entirely driven by the individual decisions [33] by evaluating patient pathways within their social, economic, and political contexts.

**Fig. 1:**
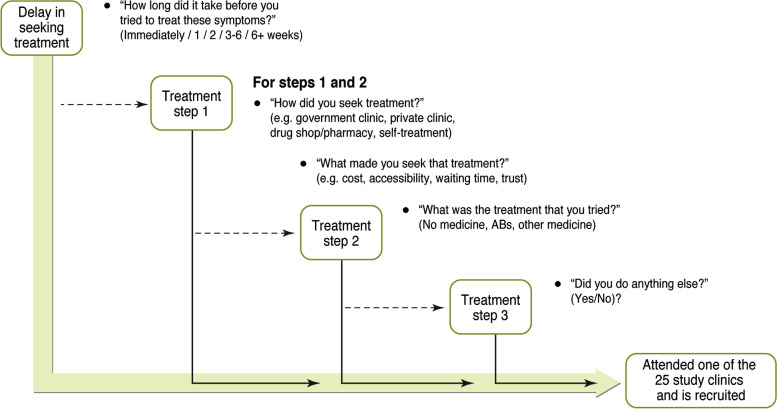
Flow of questions used to collect quantitative data on the patient treatment-seeking pathway for UTI-like symptoms

### Sample

The sample consists of 6,827 adult outpatients, aged 18 and over (or pregnant and < 18) who were recruited from healthcare facilities in Kenya, Tanzania, and Uganda, within three sites per country (Kenya: Nairobi, Nanyuki, and Makueni; Tanzania: Mwanza, Kilimanjaro, and Mbeya; Uganda: Mbarara, Nakapiripirit, and Nakasongola), between February 2019- September 2020. Full study details, including sample size considerations, inclusion and exclusion criteria are published elsewhere [29]. We recruited patients at primary and secondary facilities (levels 2–5) which were predominantly government-funded (Table S1). Clinicians identified patients with symptomatic and probable UTI for inclusion. In all sites, less than 1% of those approached declined to participate. Patients provided a mid-stream urine sample and answered a questionnaire with trained fieldworkers on treatment-seeking for UTI symptoms, AB use practices and attitudes, and sociodemographic characteristics. We excluded 219 patients who came to the recruitment clinic for non-UTI symptoms and had not attempted to treat their symptoms, leaving 6,608 patients for analysis: 3,190 (48·3%) from Tanzania, 1,757 (26·6%) from Uganda, and 1,661 (25·1%) from Kenya. The patient urine samples underwent microbiological culture, and UTI (defined by the presence of > 104 colony-forming units per millilitre (CFU/mL) of one or two uropathogens) was present in 2,264 (24%) of patients.

In-depth interviews (IDIs) were conducted 1–2 weeks after the clinic visit with a purposively selected subset of patients (*n* = 116) who had microbiologically confirmed UTI, reported longer treatment pathways or were diagnosed with a multi-drug resistant UTI pathogen. IDIs were conducted in person, at the respondents’ homes and in their primary language, using standardised topic guides, which were subsequently translated into English by fieldworkers. IDIs focused on mapping individual pathways based on the patient’s account of recent treatment-seeking action, AB use, knowledge and attitudes, and motivations for behaviours. Patient qualitative and quantitative data were linked using numeric identifiers, rather than personal details, to allow us cross-reference between types of data to understand biomedical, social, economic, and attitudinal characteristics of patients. Participants gave written informed consent. Patients were not involved in the design, or conduct, or reporting, or dissemination plans of our research. Ethical approval was obtained from National and Institutional Research Ethics Committees (see protocol) [29].

### Study variables

#### Treatment seeking behaviours

Figure 1 illustrates the structured questionnaire used to collect patient pathway information, which identified the types of providers consulted, treatments taken, and reasons for these choices.

We study three binary outcomes with a theoretical or empirical link to ABR risk identified in previous studies: [11, 34, 35]

1. Self-treatment as a first step (defined as going to a drug shop/pharmacy, seeking advice from friends or family, or using traditional or home remedies) vs seeking help at a medical facility;
2. Multi-step pathway: Having two or more steps in the pathway prior to coming to the recruitment centre vs. having fewer steps;
3. Taking ABs from any source to treat their symptoms vs not taking any ABs prior to coming to the recruitment clinic.

The last variable was derived from patients’ self-reports of the names of medicines taken during the pathway. During the interview, respondents were prompted using a drug bag or drug card developed specifically for each site [36].

#### Other variables

We included self-reported variables that might impact treatment-seeking patterns [11]. Sociodemographic factors included gender (male/female), age (categorised into < 25; 25–34; 35–44; 45–54; 55–64; 65 + years), marital status (married, never married, and other, which included cohabiting, widowed, divorced), and household size (1–2 people, 3–6 people, 7 + people). Socioeconomic status was measured by education level (no formal, primary, secondary, and tertiary), employment status (formal employment, informal employment, homemaker, not working), self-reported difficulty in meeting healthcare costs (easy, difficult, very difficult), and a within-country asset index grouped into quintiles (Table S2 for details). Healthcare factors included the level of medical facility the patient was recruited from (lower-level community health centres (levels 2–3) vs. high-level clinics or referral hospitals (levels 4 +), and whether they had previous experience of UTI (did not have UTI before, had UTI before, or did not know what UTI was). We include a binary variable indicated whether the patient had any type of health insurance. We also included indicators which measured whether patients felt that UTI symptoms were stigmatised (yes/no), which may impact treatment-seeking.

### Statistical methods

We used Sankey plots to visualise quantitative data on patient pathways, showing counts and percentages of types of providers chosen and type of treatment obtained (if any) at each step. We excluded patients without valid data on the steps considered (*n* = 230), leaving a sample of *n* = 6,378. Subsequently, we used Bayesian hierarchical logistic regression models to assess socioeconomic and attitudinal factors associated with three binary outcomes outlined above (full model specification in Supplementary Sect. 3). The models were estimated in R using the Nimble package [37]. Approximately 8% of our sample had missing values on the outcomes or covariates, which we account for within a Bayesian modelling framework. Regression models had four levels: patients were nested in 25 clinics, clinics in nine sites, and sites within three countries. Results were reported in terms of odds ratios (ORs) and 95% highest posterior density intervals (HPDI) due to the skewed posterior distribution of all independent variables. We conducted a sensitivity analysis, with the same models restricted to the patients with microbiologically confirmed UTI (reported in Supplementary material S13).

### Qualitative data analysis

Translated English-language interview transcripts were linked to quantitative data using patients IDs, and analysed using NVivo software [38]. We used iterative thematic content analysis, beginning with first-round coding based on interview questions, such as how patients sought treatment and obstacles to treatment-seeking. Subsequent rounds of in-depth coding were undertaken to identify differences and similarities in treatment-seeking pathways between patients, as well as potential contributing factors to decision-making around treatment seeking.

## Results

### Sample characteristics

Most patients (79%) were females of reproductive age, and the majority were married (Table S3). In Uganda and Tanzania, most had little or no formal education, whereas in Kenya 86% had secondary or higher education. In the pooled data, most were either in informal employment or homemakers (41% and 25% respectively), and 60% lived in households of 3–6 people. Ugandan respondents were least likely to report it was ‘easy’ to meet healthcare costs, compared to Tanzania and Kenya. Around half of respondents from Tanzania and Kenya reported they had been previously diagnosed with UTI, compared with one quarter in Uganda. Possibly related is that symptoms stigma was higher among Ugandan participants (39%) compared to those in Tanzania and Kenya (16% and 23%, respectively). Patients in Tanzania and Uganda were mostly recruited at lower-level facilities (level 2–3), whereas in Kenya most were recruited at higher-level facilities or referral hospitals (levels 4 +). Sociodemographic characteristics for the qualitative sample reflected those in the quantitative (Table S4).

### Overview of patient pathways

**Fig. 2:**
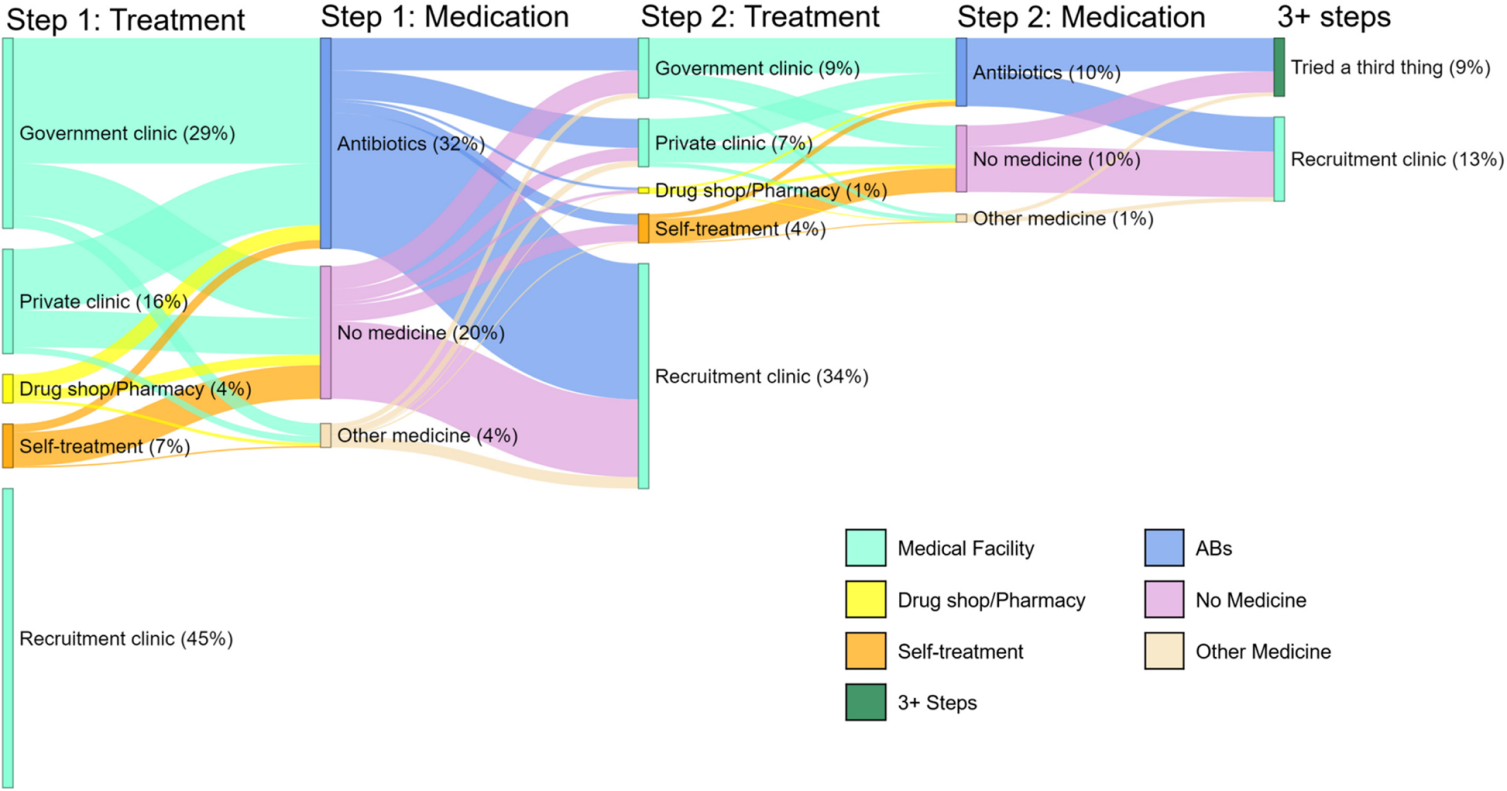
Sankey plot describing patient treatment seeking pathways for UTI-like symptoms for pooled analysis (*N* = 6,378). Figure notes: *n* = 230 were excluded from the analysis sample due to missing data on the relevant variables. Percentages might not add up to 100% due to rounding

Figure 2 illustrates the choices of provider and whether ABs were obtained for each pathway step pooling data across 3 countries; analogous country-specific plots are shown in Supplementary Figure S7. The pathways as shown are composed of a maximum of three steps. At step 1 or 2, patients are classified as going to either a government clinic, private clinic, drug shop, self-treating, or going directly to a recruitment clinic. At the third step, participants reported either trying a third option or attending the recruitment clinic. Across all countries, most patients (86%) went to medical facilities as their first step in treating UTI-like symptoms (Fig. 2). The largest group of patients (45%) went directly to a HATUA recruitment clinic, 26% visited another government-funded facility, 15% visited a private facility, 7% visited drug shops/pharmacies and 7% self-treated either with home remedies, herbs, or drugs provided by friends/relatives. Kenyan patients had the simplest pathways and were most likely to go to a recruitment centre as their first step. Visiting private clinics as a first step was more common in Tanzania and Uganda than in Kenya, whereas visits to drug shops and pharmacies were more common in Kenya than the other countries.

### Reasons for first choice of treatment

**Fig. 3:**
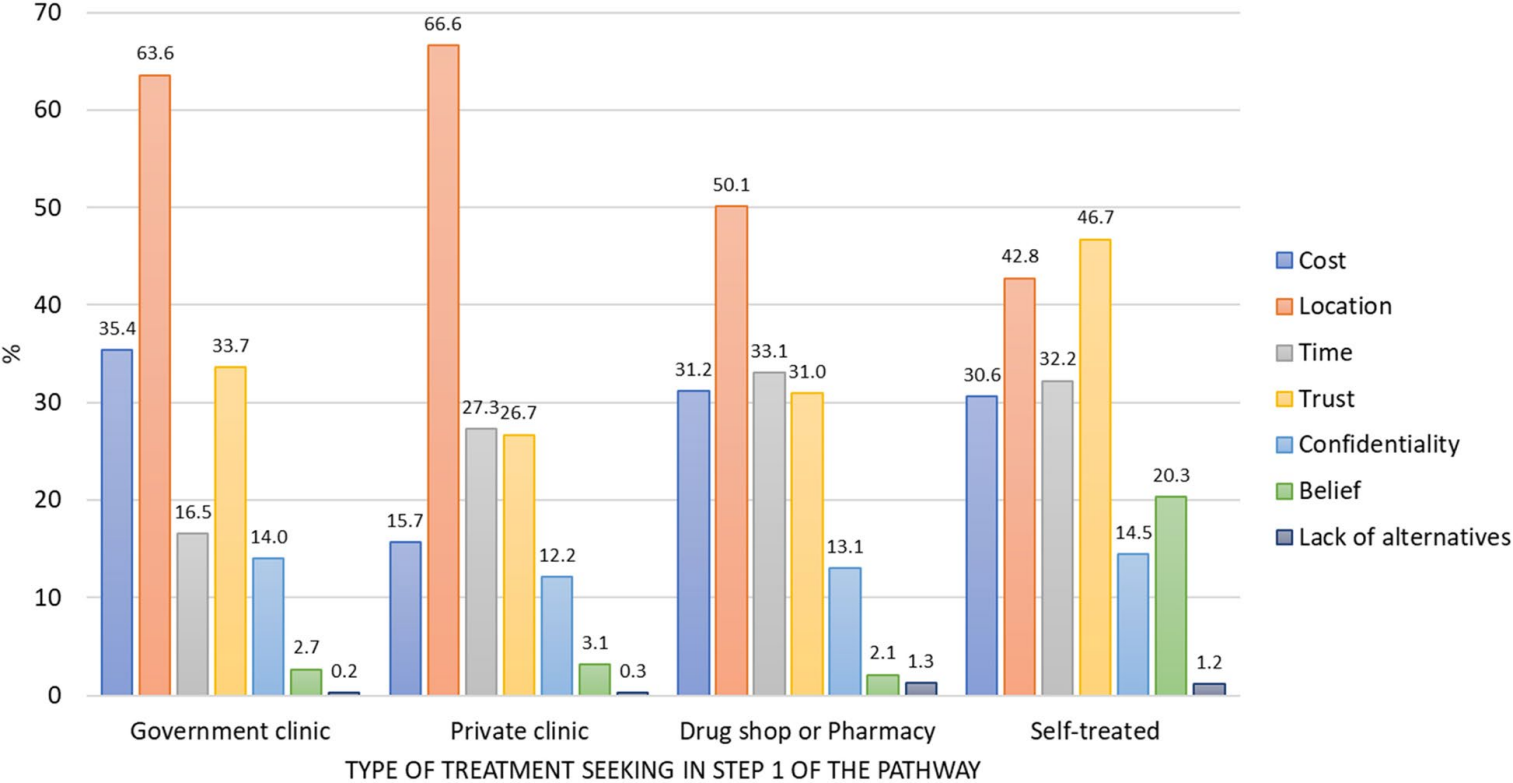
Descriptive statistics on reasons for patient’s first choice of treatment (*n* = 3,546). Notes: Includes patients who tried to treat their UTI symptoms before going to the recruitment clinic (*n* = 3,546); patients could choose multiple reasons

The reported reasons for first choice of treatment are shown in Fig. 3 (country-specific results in Fig. S8). Those who chose a government or private facility were most often motivated by location, but cost was also cited as a reason for choosing government clinics, drug sellers, and self-treatment (less so private facilities). Time was a common reason for choosing drug shops or self-treatment. Qualitative data shows that cost was often related to both location (due to transportation expenses) and time (due to loss of work), which can influence both the choice of medical facility and the decision to self-treat at step one:

“*[I live] far from the hospital or health centre, if I feel pain the first thing is to go [to the] pharmacy, there they will assist me with a drug that will ease pain at that moment. But if pain persists, I will have to go to the hospital for further treatment and advice from the doctor.*” (male patient, Mbeya, Tanzania)

‘Trust’ was the most cited reason among those using self-treatments. Qualitative accounts indicated this can be related to trusting one’s own knowledge to treat illness:

“*Normally, I first observe the condition as I take the concoction for ginger, garlic, lemon and honey. That mixture heals everything, I don’t know why it does not heal HIV/AIDS.*” (male patient, Nanyuki, Kenya)

In some cases, ‘trust’ in formal medical facilities is eroded when patients’ expectations are not met, and symptoms remain unresolved, prompting patients to favour other treatment sources. Qualitative data also highlighted that drug shops were seen as convenient alternatives to medical facilities, and that drug sellers were sometimes viewed as part of the trusted cadre of healthcare professionals. For example, one patient explained: “*I trust only medical help from doctors and nurses, so the health centres and drug shops is where I only go*” (female patient, Nakapiripirit, Uganda).

**Fig. 4:**
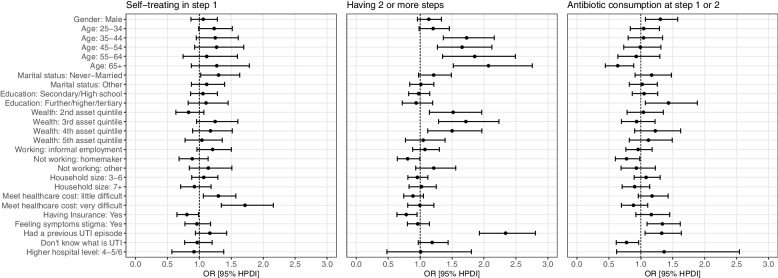
Odds ratios and 95% HPDI from adjusted logistic regression models for outcomes of self-treating in step 1, having 2 + steps in the pathway and taking ABs in the pathway (*N* = 6,608). Notes: Antibiotic consumption at step 1 or 2 outcome (*n* = 3,546) excludes patients going to the recruitment clinic as their 1^st^ step. Reference categories: Feeling symptoms stigma (‘No’) Meeting healthcare costs (‘Easy’); Had previous UTI episode (‘No’); Age (< 25 years); Education: No quals/ primary; Marital status (‘married’); Wealth quintile (1.^st^- poorest); Working (‘formal employment’); Household size (1–2 people); Hospital level (2–3)

Results from multivariable regression suggest the importance of perceived costs on decision to self-treat. After adjustment for socio-demographic variables, self-treating from a drug shop or with home remedies at step 1, versus choosing a medical facility, was more likely among patients who find it ‘a little difficult’ (OR = 1·29; 95%HPDI = 1·06, 1·57) or ‘very difficult’ (OR = 1·71; 95%HPDI = 1·35, 2·16) to meet healthcare costs, relative to those who found it ‘easy’ (Fig. 4, panel 1). However, this association was strongest in Kenya, relative to the other countries (see country specific plots, Figure S9). Having health insurance was associated with 20% reduced odds of self-treating (OR = 0.80, 95% HPDI 0.65–0.98), and this association was strongest in Tanzania (OR = 0.50, 95%HPDI 0.37, 0.71) (Figure S9).

Attempted self-treatments included consuming herbal remedies and ABs obtained in drug shops without a prescription, as shown in this pathway from a female patient in Nakapiripirit, Uganda.

Step 0, Delay: *“When I started feeling this pain I first ignored [it]”*

Step 1, Traditional Medicine: *“Later I went to [a traditional healer] where I was told that I was bewitched … The herbalist gave me some leaves for bathing … then for drinking I was given moringa, he told me to take for one month ... I felt fine for that one month, but it didn’t help me.”*

Step 2, Drug seller: “I began buying the medicine in the drug shop. I was given amoxicillin capsule and metronidazole. I bought [a] full dose that I took for 3 weeks I was told to take 2*3 a day, which I did. In addition, I stayed for some months.”

Step 3, Drug Seller: *“After some time, it started again, and I decided to go and buy more medicine which was not full dose because I didn’t have enough money to buy the medicine.*”

Step 4, Recruitment Facility: “*Then in March I met sister who… [advised me*]* to come for check-up and I was given medication for one month from [this] heath centre.”*

Here, initial self-treatment behaviour was driven by confusion: “*I just don’t know what to do*” (female patient, Nakapiripirit, Uganda). After unsuccessful treatment attempts with the traditional medicine and drug sellers, the patient reflected that for future ailments: “*I will be coming to get medication at the hospital not somewhere else since they prescribe well and give the right medication.”*

### Understanding multi-step pathways

**Fig. 5:**
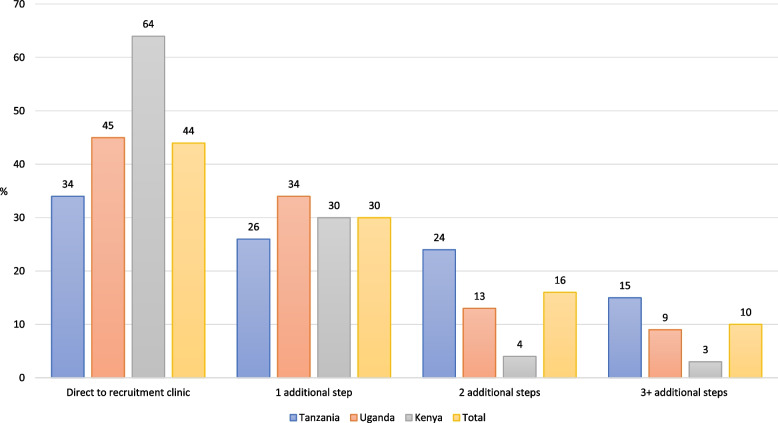
Number of pathway steps as percent of the total within each country (*N* = 6,608)

Overall, 56% of patients tried some other form of treatment before arriving at the recruitment facility; we label these as having ‘multi-step pathways’. Of these patients (*n* = 3,546), 53% took one step before the recruitment clinic (30% of the full sample), 29% had two additional steps (16% of the full sample), and 18% had three or more steps (10% of the full sample) (Fig. 5). Multi-step pathways were more common in Uganda and Tanzania than Kenya.

While some multi-step pathways did include self-treatment or drug seller visits, most began with patients seeking treatment from medical facilities, after which pathways became convoluted, often involving repeated visits to medical facilities, causing great frustration, as evident in the pathway description by a male patient from Mbarara, Uganda:

Step 1, Facility 1: *“When I got infected, I went to [facility 1] … He injected me and gave me drugs to take and told me that I had recovered. I … took the medicine but I did not recover. I remained sick.”*

Step 2, Facility 1: *“I went back and … I asked him, haven’t I healed now, he wrote for me more medicine, I went to the pharmacy and bought the drugs ... I took the medicine and I seemed to recover but pain reduced.”*

Step 3, Drug Seller: *“When I finished [the prescription], I thought I would recover but I went back to the same state. So, I looked at the place I went to buy the medicine, I went there, and they wrote for me this one.”*

Step 4, ‘Leave it’: *“[The treatment] has not worked. So, I decided to leave it for a while.”*

Step 5, Facility 2: *“When the sickness continued, I [visited another] doctor … He tested me, gave me medicine, and injected me six times ... Then he took my urine samples ... He told me that the medicine he was injecting couldn’t cure it. He then brought another type of medicine … [but] when the dose was over … I started feeling like the disease had come back. Then I wondered how I was, if I had gone everywhere and the disease was failing, now where was I to go!”*

Step 6, Recruitment facility: *“The doctor … had called a specialist who would test my urine … and give me proper treatment … I decided to go there, and they got my urine samples.”*

We conducted analysis on a subset of data collected after January with data on diagnostic use in the pathway. This showed that 73% had had their urine sample tested (although it is unknown what kind of test); and that urine testing was more common at private than government facilities (Fig. S12).

Having a multi-step pathway was more common in patients older than 35 years, in middle asset quintiles, who had health insurance, and those with a previously diagnosed UTI (Fig. 4, panel 2). Stigma around UTI symptoms was associated with longer pathways, dependant on context. In Uganda, where 39% of patients felt stigma, it was associated with higher odds of having a multi-step pathway (OR = 1·43; 95%HPDI = 1·06, 1·90) (Figure S10). By contrast, in Tanzania, where only 16% of patients reported UTI symptoms stigma, this was associated with simpler pathways. IDIs also suggested that in some contexts, stigma drove treatment choices. For example, Kenyan patients discussed choosing private or distant medical facilities to avoid being recognized by members of the community. Private clinics were also favoured because treatment was faster, meaning patients could avoid having to explain absences from work: “*If I went to a public hospital, I could have taken a long time, and my friends at the market could have wanted to know what my problem was and this way I tried my level best to hide it”* (female patient, Makueni, Kenya).

### Antibiotic consumption during the pathway

Increasing the number of pathway steps provided more opportunities for AB consumption. Among patients with multi-step pathways, nearly half (48%) reported taking ABs at step one, and 42% of those with a second step took ABs (Fig. 2). As Fig. 2 shows, most ABs were consumed after visits to medical facilities. The most common ABs taken were amoxicillin and ciprofloxacin (see Table S5), and some ABs taken were not recommended for treating UTI symptoms (e.g., doxycycline). AB consumption was higher among those with UTI symptoms stigma (OR = 1·34; 95%HPDI = 1·09, 1·62), those who had been previously diagnosed with UTI (OR = 1·33; 95%HPDI = 1·07, 1·63), and those who had higher-level educational qualifications (OR = 1·43; 95%HPDI = 1·07, 1·89) (Fig. 4, panel 3); AB consumption was lower among those aged 65 + (OR = 0·63; 95%HPDI = 0·44, 0.88), those who don’t know what a UTI is (OR = 0.77, 95%HPDI 0.61,0.96), and among homemakers, relative to those in formal employment (OR = 0.77, 95% HPDI 0.60,0.98).

Qualitative data enriched the picture with stories of repeat prescriptions for the same drugs procured from multiple visits to medical centres and drug sellers, evident in the pathway of a female patient from Moshi, Tanzania:

Step 1, Private Facility: *"They started with amoxicillin. Then they gave me ampiclox. Then they gave me amoxiclav. But symptoms persisted."*

Step 2, Government Facility 1: *"Then I … went to Kilimanjaro hospital, and there I still had UTI … They gave me amoxiclav."*

Step 3, Government Facility 2: *“Then I went to [another] hospital, they also gave me amoxiclav… and they said that was a strong drug."*

Step 4, Recruitment Facility: *"I went there because I didn’t get a cure. And I had two problems. The biggest one was ulcers, and I still had UTI symptoms… [the doctor] suggested the tests I needed to take. I agreed and they tested me … [then] he told me that he is giving me a seven-day dose [of amoxiclav].”*

This patient further expressed concern that her insurance coverage impacted the drugs she was given: “*Sometimes a doctor may prescribe same drugs that you have used before and when you ask him he says it is the insurance*” (female patient, Moshi, Tanzania). Quantitative data corroborated this picture. In country-specific quantitative models (Figure S11), having health insurance was associated with higher chance of antibiotic use (OR = 1.64, 95%HPDI 1.20, 2.20) in Tanzania, but not the other countries. In these settings, visits to medical facilities and drug sellers often go hand in hand, due to stockouts in public health facilities:

“*You find yourself not having money, [and at the hospital] you are just given a prescription and told to buy drugs. If you don’t have money you won’t buy, until you get money that is when you will buy”* (female patient, Mwanza, Tanzania).

This contributed to decisions to go directly to drug sellers to save the cost of medical consultation.

## Discussion

Using mixed-methods data, we show that patient pathways in East Africa for a common infection (UTI) are often convoluted, involving reiterative steps and different healthcare providers. Such complexity was not driven by individual choice; patients were struggling to get care that worked in a confusing, hybrid healthcare landscape riddled with structural constraints. Our findings support others which stress the importance of location, cost, and time in treatment decision making, [11, 39], but we show these are contextualised by community factors such as stigma, illness behaviour, and local understandings of illness which are in turn conditioned by wider socioeconomic, geographic, and healthcare structures. These patterns also need to be placed in the context of struggles to access formal healthcare alongside relative ease of access to antibiotics at pharmacies and drug shops in the region [39, 40].

Even though patients predominantly chose what might be regarded as clinically ‘optimal’ paths, i.e., attending medical facilities, this often did not resolve their symptoms. The causes of these treatment failures in healthcare settings deserve further investigation. Over three quarters of the patients in the study did not have a microbiologically confirmed UTI, so empirical treatment for this infection was not likely to resolve their symptoms. Some of those with microbiologically confirmed UTI will have forms of drug resistance which make their UTI more difficult to treat. Improvements in access to diagnostic capabilities in LMICs [41] could detect forms of infection and drug resistance and guide appropriate treatments at earlier stages in the treatment journey. One area to address could be better ways to record medical history so that clinicians understand what drugs have been taken and why. Another issue may be equitable access to appropriate treatments: around two thirds of those attending clinics ended up not taking any medicines, and our data suggest this may be due to stockouts at clinics and prohibitive costs of medicine [11, 13]. Future studies should address clinic-related factors in depth, including the patient-doctor consultation, medical records keeping and information flows, and other barriers that prevent medical staff and patients from following best practice advice around ABs. Given that most patients sought care at public health facilities, this underlines the importance of understanding limitations in healthcare systems and infrastructure to address the threat of AMR.

Patients’ treatment choices were often motivated by time and financial constraints, which can lead to systematically different pathways among the poorest and the richest patients. More educated and wealthier patients were both more likely to consume ABs and have multi-step pathways, corroborating findings from other studies [10, 42]. As described elsewhere, [13] wealthier subgroups may be also more likely to choose private facilities and buy medicines from drug sellers to save time. On the other hand, poorer subgroups or those struggling to afford healthcare are more likely to self-treat and struggle to afford appropriate AB treatment.

This study has some limitations. The linked quantitative–qualitative sample is representative of the patient population attending public facility outpatient services for UTI symptoms at study sites (future consortium papers will address community members not attending clinics). We repeated the analysis restricted to patients with microbiologically confirmed UTI and the same pattern of effects was seen (Figure S13). We would also recommend repeating the study with a focus on other symptoms for other common conditions that prompt antibiotic use (e.g. upper respiratory tract infections), because they potentially have different levels of stigma and are confused with other conditions such as COVID-19 [21]. As mentioned above, further emphasis on clinic-related factors, such as diagnostics, medicine availability, prescribing patterns is warranted. Given the self-reported nature of treatment-seeking behaviour and its predictors, we cannot rule out reporting bias.

## Conclusion

This study has taken a patient-centric perspective, but our results suggest that treatment-seeking is never an individuated behaviour; actions are influenced by situational constraints and are contextually dependent. Thus, AMR should be considered a system rather than a set of individual actions. Complex treatment pathways are likely related to various individual and structural factors, but another important driver is likely to be ABR itself. As ABR continues to evolve, the cyclical treatment attempts we observed here for UTI-like illness will become more common, reflecting the vicious socio-biological cycle of ABR. Drug resistance means treatment attempts are more likely to fail, thus fuelling ABR by necessitating further AB treatments. In many LMICs, there are key structural weaknesses which facilitate this vicious cycle: including (but not limited to) under-resourced public healthcare, insufficient diagnostic capacity, and ample opportunities to purchase ABs without prescription. Thus, we advocate that attention be paid towards addressing these upstream factors which drive both ABR and complex patient pathways.

## Supplementary Information

**Additional file 1. **

## Data Availability

The datasets generated and/or analysed during the current study are not publicly available due to ethical and data access agreements with individual country ethical boards but are available from the corresponding author on reasonable request.

## Abbreviations

ABR: Antibacterial resistance
ABs: Antibiotics
HATUA: The Holistic Approach to Unravelling Antibacterial Resistance Consortium
UTI: Urinary tract infection
LMIC: Low- and middle-income country
IDIs: In-depth interviews
OR: Odds Ratio
HDPI: Highest posterior density interval
