# Supplementary material for: Unravelling patient pathways in the context of antibacterial resistance in East Africa
Source: BMC Infect Dis. 2023 Jun 19;23:414. doi: 10.1186/s12879-023-08392-9 (PMC10278291; doi:10.1186/s12879-023-08392-9)
Supplement: Supplementary file 1 — Additional file 1. [file 12879_2023_8392_MOESM1_ESM.docx]

# **Supplementary Material**

**Contents**

[**1: HATUA patient recruitment: sites and healthcare facilities 2**](#_Toc92698420)

[**2: Developing an asset index 3**](#_Toc92698421)

[**3: Statistical Appendix: Bayesian Hierarchical Modelling 5**](#_Toc92698422)

[**4: Supplementary material for Results 13**](#_Toc92698423)

## **1: HATUA patient recruitment: sites and healthcare facilities**

HATUA patient recruitment took place in 9 sites (three each in Kenya, Tanzania, and Uganda). Healthcare facilities were chosen across a number of primary and secondary care levels.

Table S1. Patient recruitment sites in Kenya, Tanzania, and Uganda

| Country/site | Number of facilities (TOTAL) | Source of funding | Levels recruited from^1^ |
| --- | --- | --- | --- |
| **Kenya** |  |  |  |
| Makueni | 1 | Public | 5 |
| Nairobi | 4 | Public and private | 3-5, National |
| Nanyuki | 1 | Public | 4 |
| **Tanzania** |  |  |  |
| Kilimanjaro/Moshi | 3 | Public and private | 2,3, and 5 |
| Mbeya | 2 | Public and private | 3 and 4 |
| Mwanza | 5 | Public and private | 2,3, and 5 |
| **Uganda** |  |  |  |
| Mbarara | 3 | Public | 3 and 5 |
| Nakapiripirit | 3 | Public | 2 and 3 |
| Nakasongola | 3 | Public and private | 3 and 4 |

^1^ Levels of facilities are identified in each country following the Kenya Health Policy 2014-2013 (<http://publications.universalhealth2030.org/uploads/kenya_health_policy_2014_to_2030.pdf>); Tanzanian Health Sector Strategic Plan (<http://www.tzdpg.or.tz/fileadmin/documents/dpg_internal/dpg_working_groups_clusters/cluster_2/health/Key_Sector_Documents/Induction_Pack/Final_HSSP_IV_Vs1.0_260815.pdf>), and the Ugandan Hospital and Health Centre IV census survey (<https://www.who.int/healthinfo/systems/SARA_H_UGA_Results_2014.pdf>).

## **2: Developing an asset index**

We developed an asset index as a proxy measure for the socioeconomic status of each patient, commonly used in LMIC survey analysis, including the Demographic and Health Surveys (DHS). An asset index provides an alternative measure to income which tends to be more stable over time and operates as a relative measure of poverty. It has been recommended to develop an asset index rather than measure each asset variable separately.[1] Previous studies have adopted Principal Components Analysis to generate an asset index, but this method has faced some criticism, particularly where it involves the inclusion of infrastructure-based variables that may impact rich-poor differences.[2] Instead, we used an exploratory factor analysis[3, 4] because we include some variables specifically related to infrastructure.

Data on assets were collected in the HATUA standardised questionnaire. We included 23 binary variables which covered ownership of durable household goods, and access to water and sanitation (listed in Table S2). To reflect the relative nature of wealth and the spatial differences covered in our study, we derived an asset index for each country separately. The Kaiser-Meyer-Olkin (KMO) was undertaken to ensure the appropriateness of the data for each index. The KMO for each index was over 0·5 (Tanzania: 0·67; Uganda: 0·75; Kenya: 0·62) and deemed suitable for factor analysis.[5] To create an index, only one factor is extracted from the data for each country. An oblique factor rotation was chosen as it is produces factors that are correlated.[5] The factor loadings varies by country. Along with the index variables, Table S2 presents the factor loadings for each country’s asset index along with the respective Cronbach alpha, which is used to test scale reliability.[6] The resulting factor is a continuous variable scoring each patient’s level of wealth from lowest to highest, which we divided it into quintiles.

**Table S2. Factor loadings for each country’s asset index**

|  | **Tanzania** | | **Uganda** | | **Kenya** | |
| --- | --- | --- | --- | --- | --- | --- |
| Asset variable | Factor scores | Alpha | Factor scores | Alpha | Factor scores | Alpha |
| Electricity | 0·29 | 0·78 | 0·57 | 0·80 | ·· | 0·76 |
| Motor | 0·27 |  | 0·28 |  | ·· |  |
| Fishboat | ·· |  | ·· |  | ·· |  |
| TV | 0·43 |  | 0·48 |  | ·· |  |
| Radio | 0·29 |  | 0·37 |  | ·· |  |
| Computer | 0·27 |  | ·· |  | 0·25 |  |
| Refrigerator | 0·43 |  | 0·29 |  | 0·42 |  |
| Smart phone | ·· |  | 0·29 |  | ·· |  |
| Older mobile phone | · |  | 0·38 |  | ·· |  |
| Beds | 0·28 |  | 0·45 |  | ·· |  |
| Tools | ·· |  | ·· |  | ·· |  |
| Herds of animals | ·· |  | -0·27 |  | ·· |  |
| Protected private washing water source | 0·90 |  | 0·79 |  | 0·91 |  |
| Protected public washing water source | -0·79 |  | -0·75 |  | -0·87 |  |
| Unprotected private washing water | ·· |  | ·· |  | ·· |  |
| Unprotected public washing water source | 0·90 |  | ·· |  | ·· |  |
| Protected private drinking water | -0·79 |  | 0·81 |  | 0·92 |  |
| Protected public drinking water | ·· |  | -0·77 |  | -0·87 |  |
| Unprotected private drinking water | ·· |  | ·· |  | ·· |  |
| Unprotected public drinking water | ·· |  | ·· |  | ·· |  |
| Private flush | 0·56 |  | ·· |  | 0·64 |  |
| Public flush | -0·41 |  | ·· |  | -0·55 |  |
| Pit latrine | ·· |  | 0·50 |  | ·· |  |

Factor loadings greater than 0·25 shown

## **3: Statistical Appendix: Bayesian Hierarchical Modelling**

We estimated the posterior distributions of the model parameters using Markov chain Monte Carlo (MCMC) algorithms implemented in the BUGS language through the NIMBLE package[7] in R Studio. Given the lack of quantitative studies describing patient treatment-seeking pathways in East African populations from which to draw priors, so we had little information from which to draw informative priors. To avoid possible bias from missing data, we estimated the models on the full sample (N=6,608) with some incomplete values on the covariates and outcomes (n=549). Five chains were initiated at different start values, where each chain ran for 100,000 iterations, with a burn-in (warm-up) of 20,000 iterations. Convergence was assessed through visual inspection of trace plots (Figures S1, S2, and S3). Trace plots were also used to guide our choice of iterations and burn-in time.

All models followed a similar multi-level structure, expressed as:

$$\text{Misuse}_{i}\sim\text{Bernoulli}\left( \pi_{i} \right)$$

$$log\left( \frac{\pi_{i}}{1-\pi_{i}} \right)=\boldsymbol{\beta X}_{i}\boldsymbol{+}{\beta clinic}_{\boldsymbol{h}_{\boldsymbol{i}}}$$

 where

$${\beta clinic}_{\boldsymbol{h}}\boldsymbol{\sim N}\left( {\beta site}_{\boldsymbol{s}_{\boldsymbol{h}}}\boldsymbol{,}\boldsymbol{\sigma}_{\boldsymbol{h}}^{\boldsymbol{2}} \right)$$

for each clinic, h.

$${\beta site}_{\boldsymbol{s}}\boldsymbol{\sim N}\left( {\beta country}_{\boldsymbol{c}_{\boldsymbol{s}}}\boldsymbol{,}\boldsymbol{\sigma}_{\boldsymbol{s}}^{\boldsymbol{2}} \right)$$

for each site, s, and

$${\beta country}_{\boldsymbol{c}}\boldsymbol{\sim N}\left( \boldsymbol{0,}\boldsymbol{\sigma}_{\boldsymbol{c}}^{\boldsymbol{2}} \right)$$

for each country, c.

The variances each having a prior $\sigma_{X}^{2}\sim exp\left( 1 \right)$. Coefficients, $\beta$, associated with covariates in the fixed effects are given a normal prior distribution with standard deviation 2·3 to give a relatively flat distribution after an inverse logit transformation. Missing data are given priors based on the empirical distributions seen in complete cases. The results of these analyses are presented as highest posterior density intervals throughout.

**Figure S1: Trace plots for outcome 1: self-treatment at step 1**


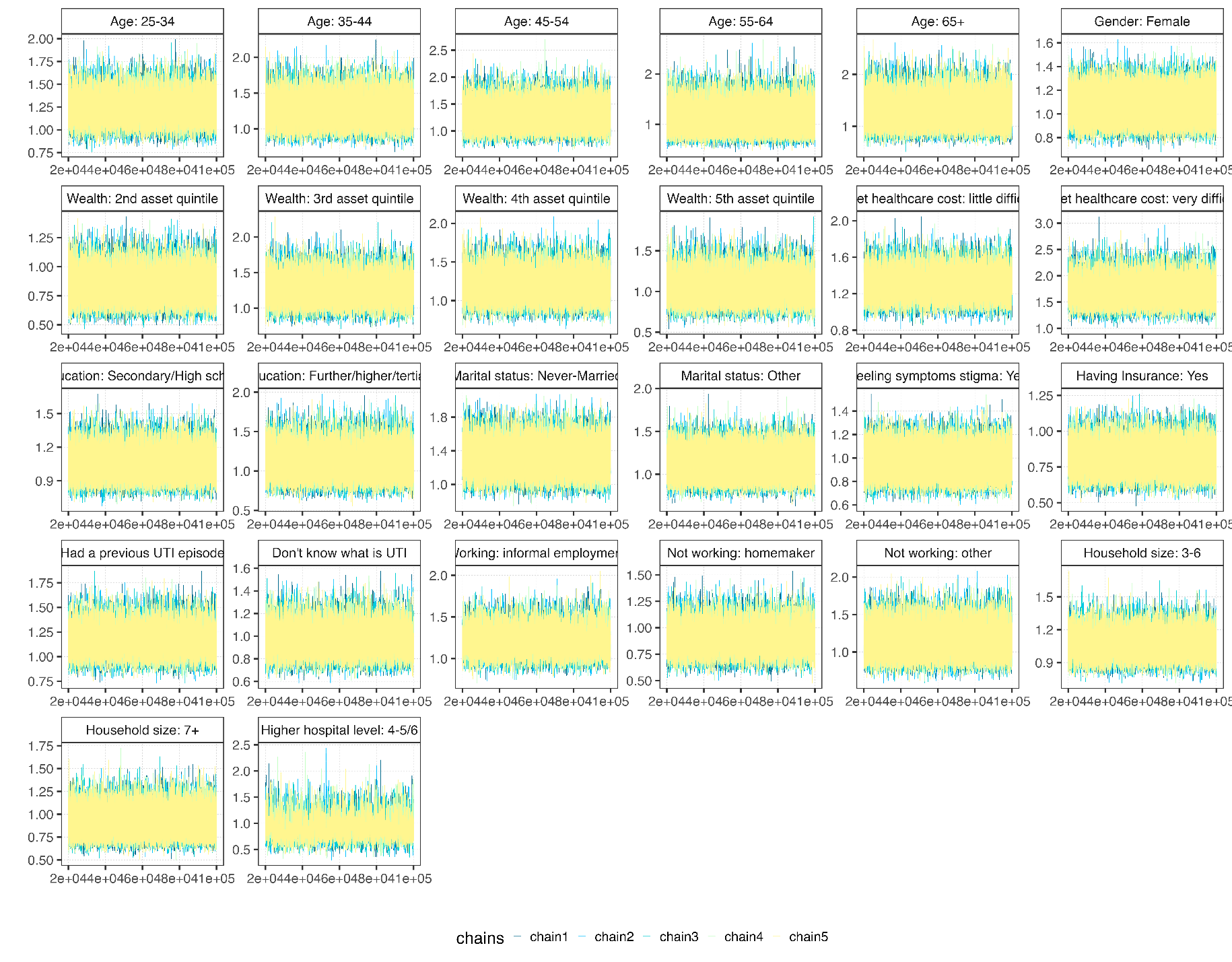


**Figure S2: Trace plots for the outcome 2: having 2 or more steps in the pathway**


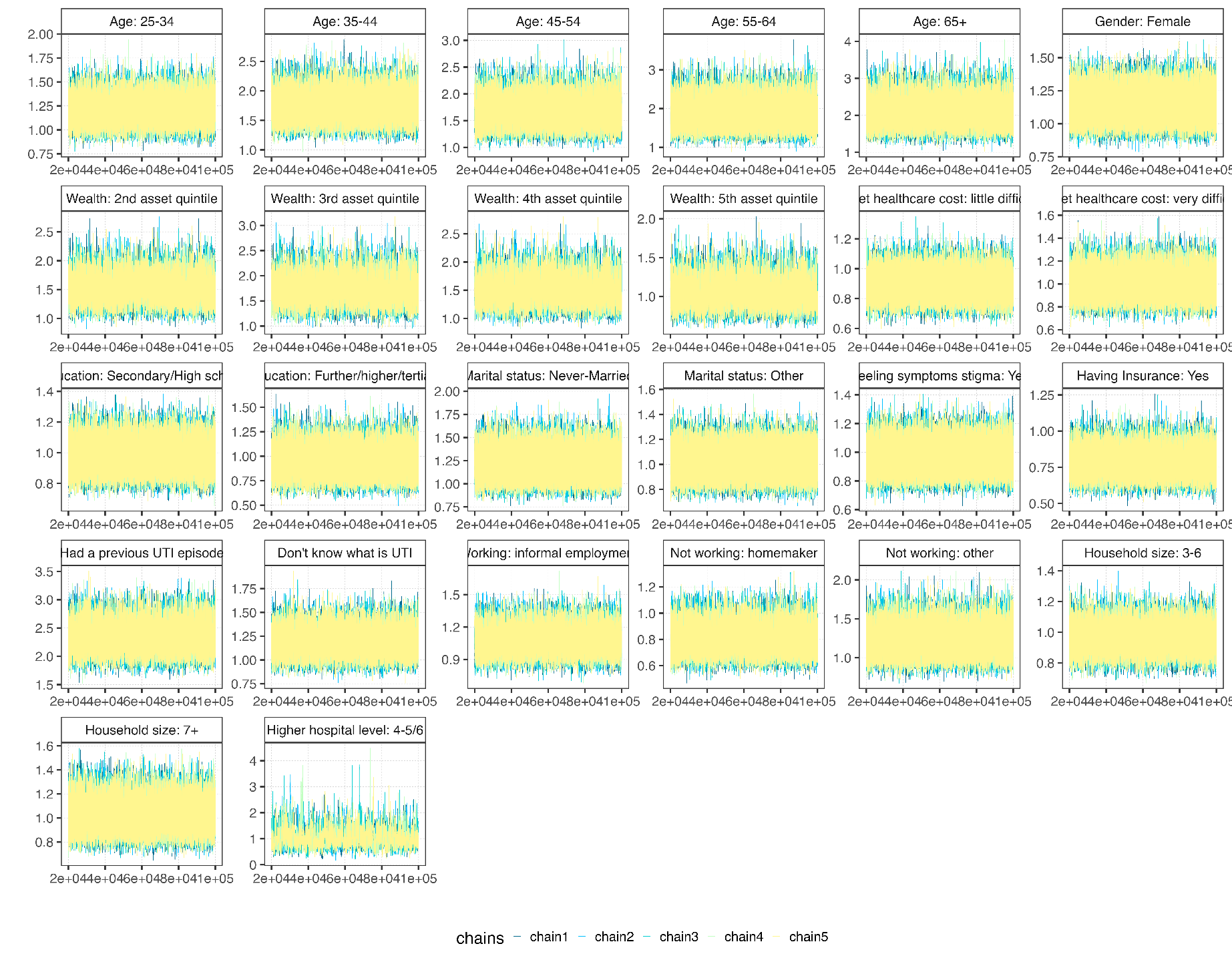


**Figure S3: Trace plot for outcome 3: antibiotic consumption at step 1 or 2**


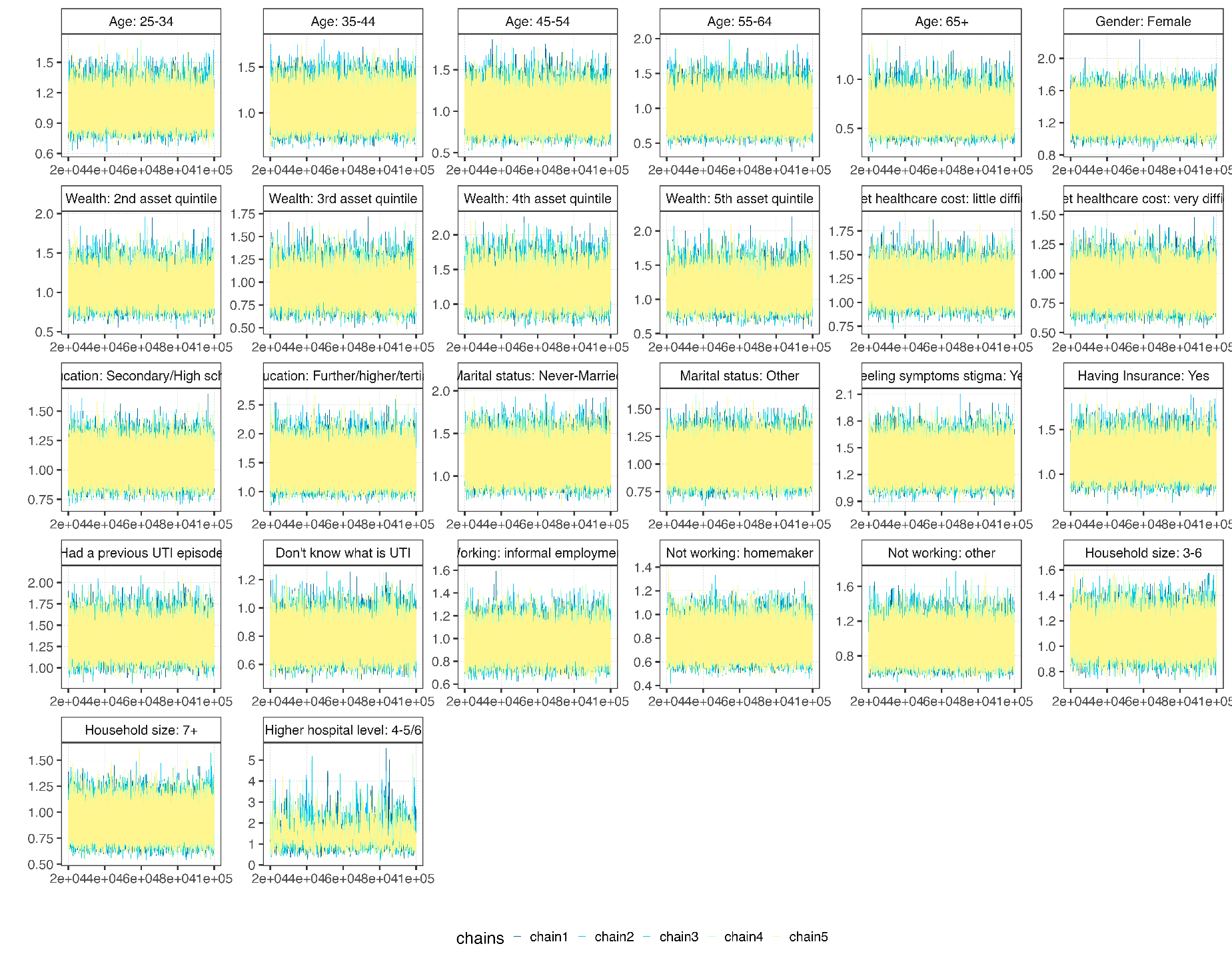


**Figure S4: Posterior distribution plots for outcome 1: self-treatment at step 1**
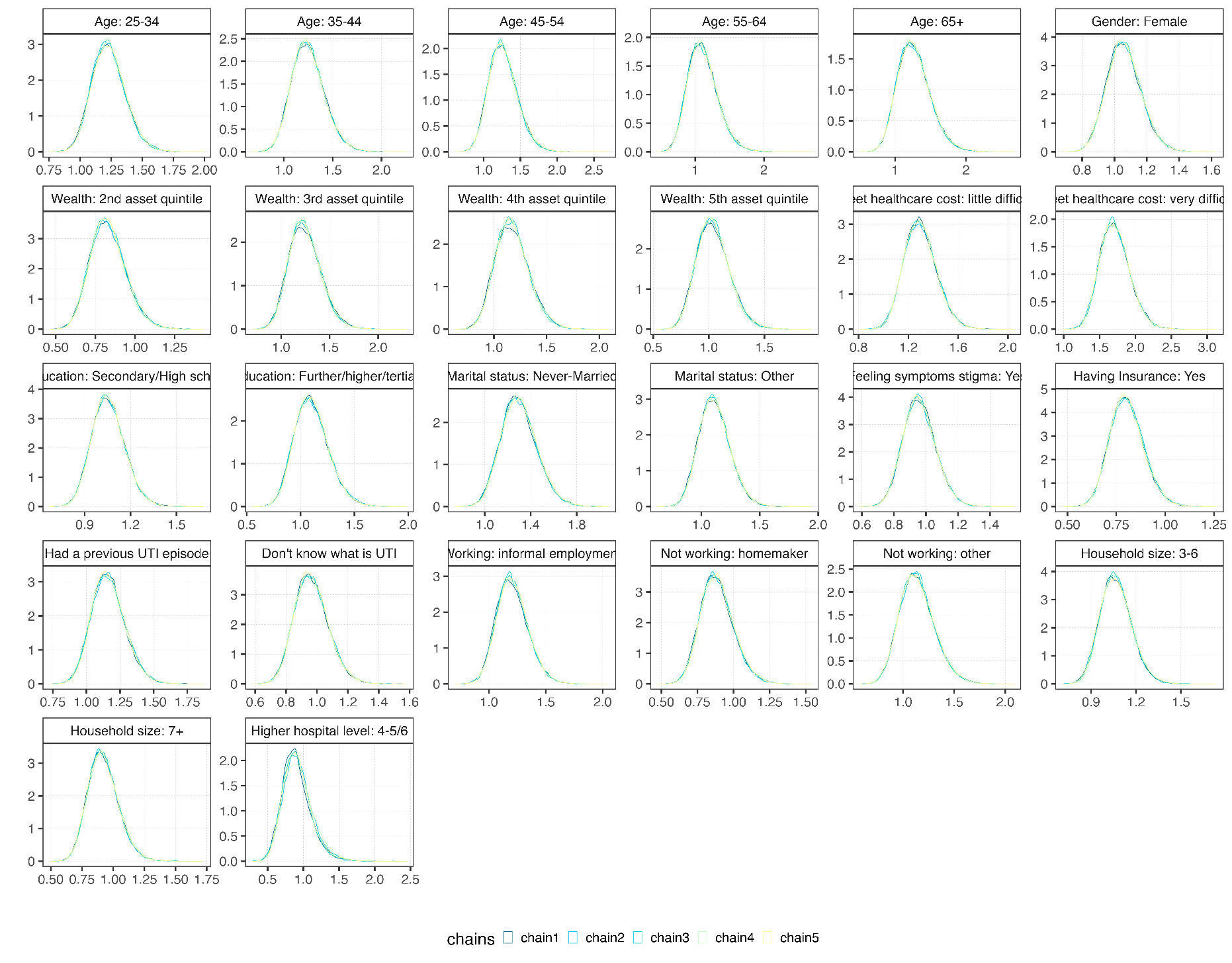


Figure S5: Posterior distribution plots for outcome 2: having 2 or more steps in the pathway


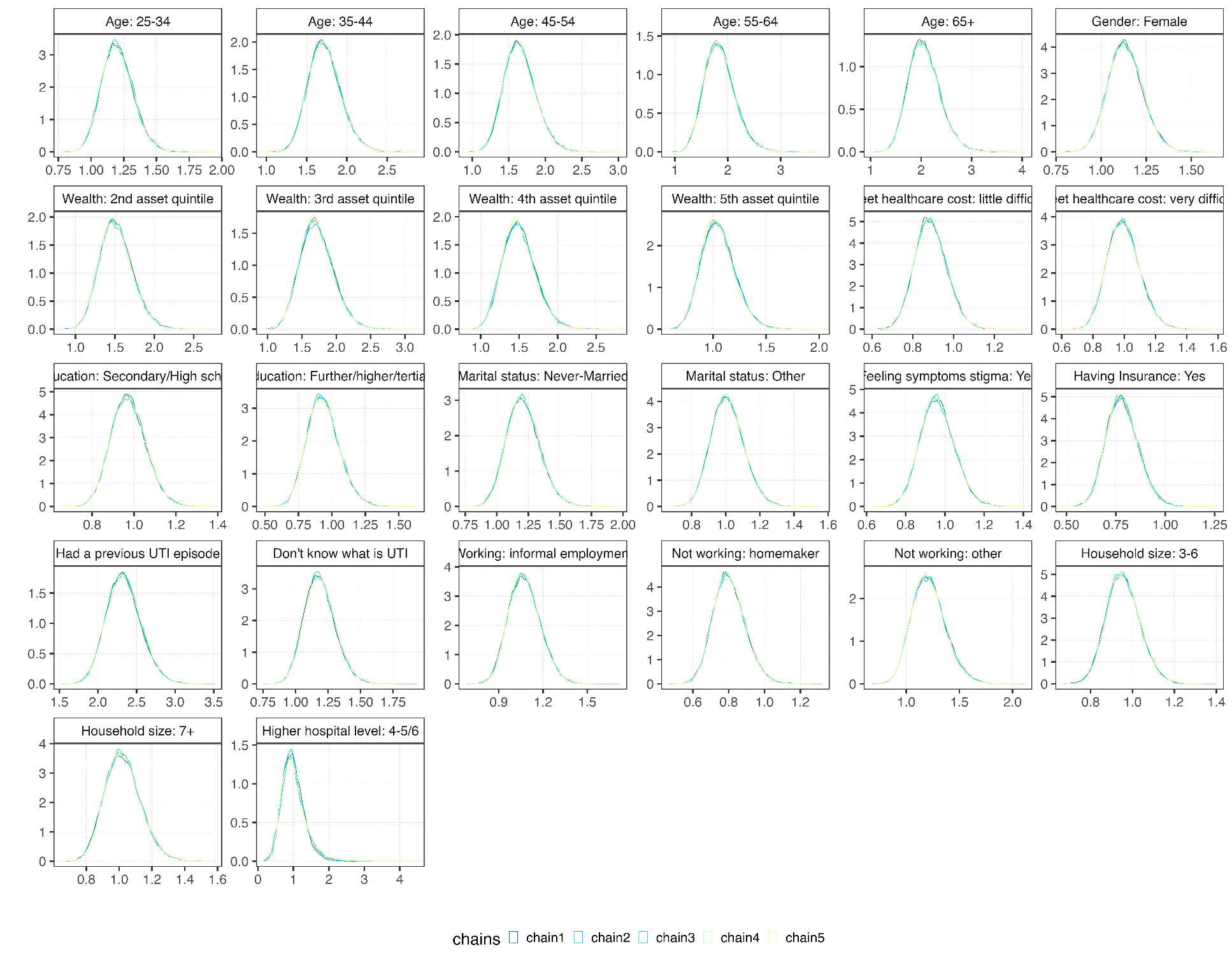


Figure S6: Posterior distribution plots for outcome 3: AB consumption at step 1 or 2


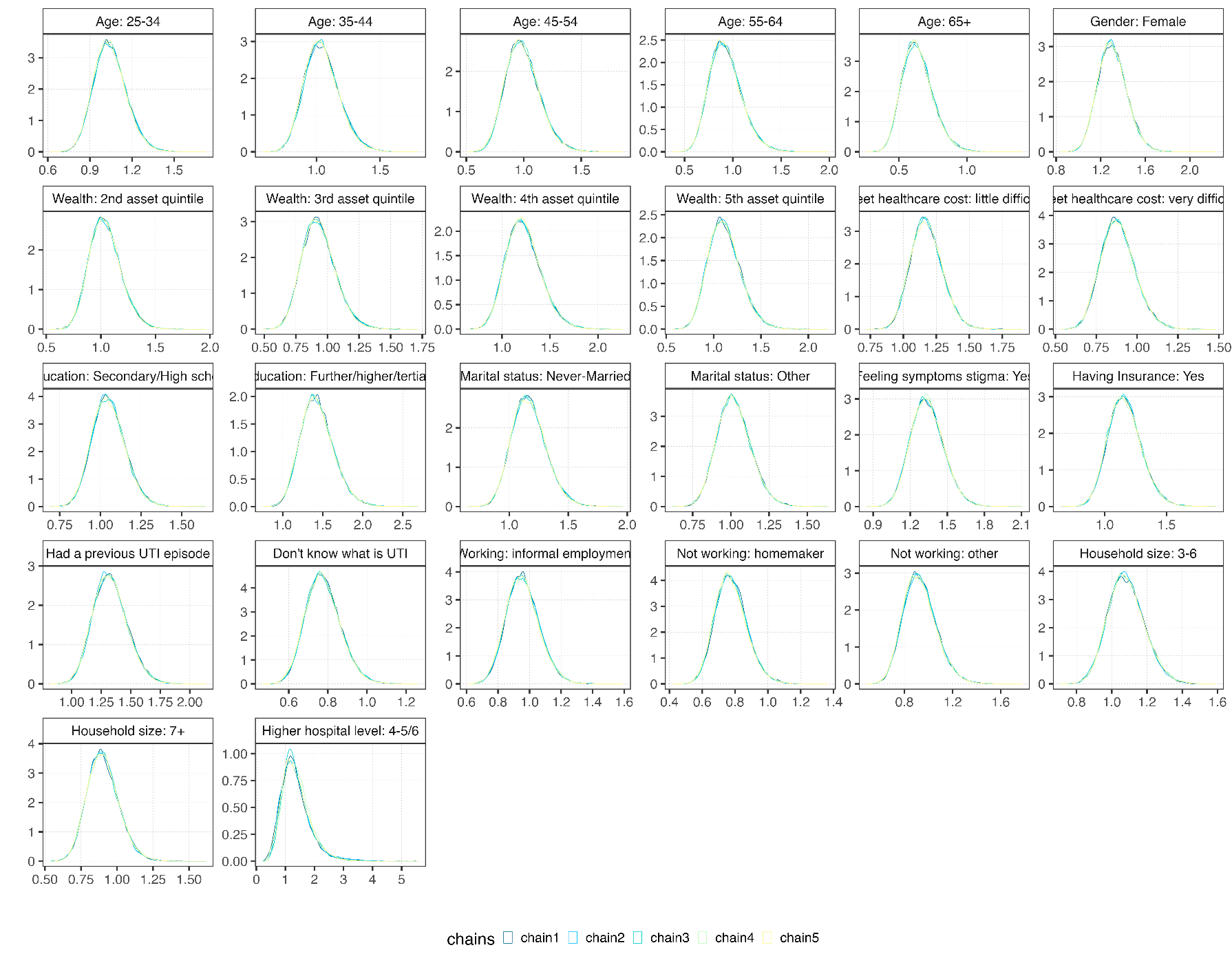


## **4: Supplementary results material**

Table S3. Characteristics of the quantitative sample (n=6608)

|  | | Tanzania %  N=3190 | Uganda %  N=1757 | Kenya %  N=1661 | Total %  N=6608 |
| --- | --- | --- | --- | --- | --- |
| Gender | Male | 26·9 | 15·9 | 16·4 | 21·3 |
|  | Female | 73·1 | 84·1 | 83·6 | 78·7 |
| Age | <25 | 22 | 33·4 | 27·3 | 26·4 |
|  | 25-34 | 26·2 | 32 | 47·3 | 33·1 |
|  | 35-44 | 15·3 | 17 | 15·8 | 15·9 |
|  | 45-54 | 12 | 9·8 | 5·2 | 9·7 |
|  | 55-64 | 8·9 | 4 | 1·5 | 5·7 |
|  | 65+ | 14·6 | 3·5 | 2·5 | 8·6 |
|  | Missing | 1 | 0·3 | 0·4 | 0·7 |
| Marital status | Married | 70·9 | 65·9 | 72·7 | 70 |
|  | Never married | 16 | 10·5 | 22·9 | 16·3 |
|  | Other: cohabiting, divorced, widowed | 13 | 23·6 | 4·3 | 13·6 |
|  | Missing | 0·1 | 0 | 0·1 | 0·1 |
| Education | No education or primary school | 66·8 | 71·4 | 14·4 | 54·9 |
|  | Secondary/  high school | 24·5 | 19·2 | 55·1 | 30·8 |
|  | Any further/ higher education, university | 8·5 | 9·4 | 30·5 | 14·3 |
|  | Missing | 0·1 | 0 | 0·1 | 0·1 |
| Wealth asset quintile  (1: poorest to 5: wealthiest) | 1 | 20·8 | 20·4 | 19·6 | 20·4 |
|  | 2 | 18 | 20 | 20·3 | 19·1 |
|  | 3 | 20·4 | 20·3 | 19·6 | 20·2 |
|  | 4 | 20·9 | 19·8 | 20·5 | 20·5 |
|  | 5 | 19·7 | 19·1 | 19·5 | 19·5 |
|  | Missing | 0·3 | 0·5 | 0·5 | 0·4 |
| Working status | Formal employment | 23·6 | 13·7 | 24·6 | 21·2 |
|  | Informal employment | 36·1 | 56 | 33·9 | 40·8 |
|  | Homemaker | 25·5 | 22·7 | 26·7 | 25 |
|  | Not working | 13·5 | 7·7 | 14·3 | 12·2 |
|  | Missing | 1·4 | 0·1 | 0·5 | 0·8 |
| Household size | 1-2 | 20·3 | 19·5 | 28·8 | 22·2 |
|  | 3-6 | 58·1 | 56·1 | 68·8 | 60·3 |
|  | 7+ | 21 | 24·4 | 1·6 | 17 |
|  | Missing | 0·6 | 0·1 | 0·8 | 0·5 |
| Ability to meet healthcare costs | Easy | 47·6 | 15·2 | 37·5 | 36·4 |
|  | A little difficult | 33 | 50·6 | 50·5 | 42·1 |
|  | Very difficult | 17·1 | 34 | 11·7 | 20·2 |
|  | Missing | 2·4 | 0·2 | 0·3 | 1·3 |
| Previous diagnosed with UTI | No | 15·4 | 24·9 | 18·3 | 18·7 |
|  | Yes | 50·3 | 24·7 | 51·7 | 43·8 |
|  | Don’t know | 32·9 | 50·2 | 27·9 | 36·2 |
|  | Missing | 1·4 | 0·2 | 2·2 | 1·3 |
| Symptoms stigma | No | 83·8 | 59·1 | 73·6 | 74·7 |
|  | Yes | 15·6 | 39·3 | 23·3 | 23·8 |
|  | Missing | 0·6 | 1·6 | 3·1 | 1·5 |
| Health Insurance | No | 67.4 | 99.6 | 45.2 | 70.4 |
|  | Yes | 32.6 | 0.4 | 54.8 | 29.6 |
| Recruitment clinic level | Lower level (2-3) | 66·2 | 78·5 | 21·4 | 58·2 |
|  | Higher level (4-6) | 33·8 | 21·5 | 78·6 | 41·8 |

Table S4: Description of the qualitative sample (n=116)

|  | | **Tanzania**  **n=31(%)** | | **Uganda**  **n=62(%)** | | **Kenya**  **n=23 (%)** | | **Total**  **n=116 (%)** | |
| --- | --- | --- | --- | --- | --- | --- | --- | --- | --- |
| **Gender** | Female | 19 | (61) | 55 | (89) | 15 | (65) | 89 | (77) |
|  | Male | 12 | (39) | 7 | (11) | 8 | (35) | 27 | (23) |
| **Age** | <35 | 9 | (29) | 39 | (63) | 12 | (52) | 60 | (52) |
|  | 35+ | 22 | (71) | 23 | (37) | 11 | (48) | 56 | (48) |
| **Wealth asset quintiles (1: poorest to 5: wealthiest)** | 1 | 5 | (16) | 20 | (32) | 3 | (13) | 28 | (24) |
|  | 2 | 1 | (3) | 10 | (16) | 6 | (26) | 17 | (15) |
|  | 3 | 4 | (13) | 21 | (34) | 10 | (43) | 35 | (30) |
|  | 4 | 12 | (39) | 7 | (11) | 3 | (13) | 22 | (19) |
|  | 5 | 9 | (29) | 4 | (6) | 1 | (4) | 14 | (12) |
| **Working status** | Formal employment | 5 | (16) | 4 | (6) | 3 | (13) | 12 | (10) |
|  | Informal employment | 13 | (42) | 43 | (69) | 9 | (39) | 65 | (56) |
|  | Homemaker | 9 | (29) | 13 | (21) | 5 | (22) | 27 | (23) |
|  | Not working-other | 4 | (13) | 2 | (3) | 6 | (26) | 12 | (10) |
| **Recruitment clinic level** | Lower level (2-3) | 18 | (58) | 49 | (79) | 3 | (13) | 70 | (60) |
|  | Higher level (4-6) | 13 | (42) | 13 | (21) | 20 | (87) | 46 | (40) |

**Figure S7: Sankey plots describing the patient pathway for UTI-like symptoms in a)Tanzania (n=3,101), b) Uganda (n=1,756), and c) Kenya (n=1,521)**

**
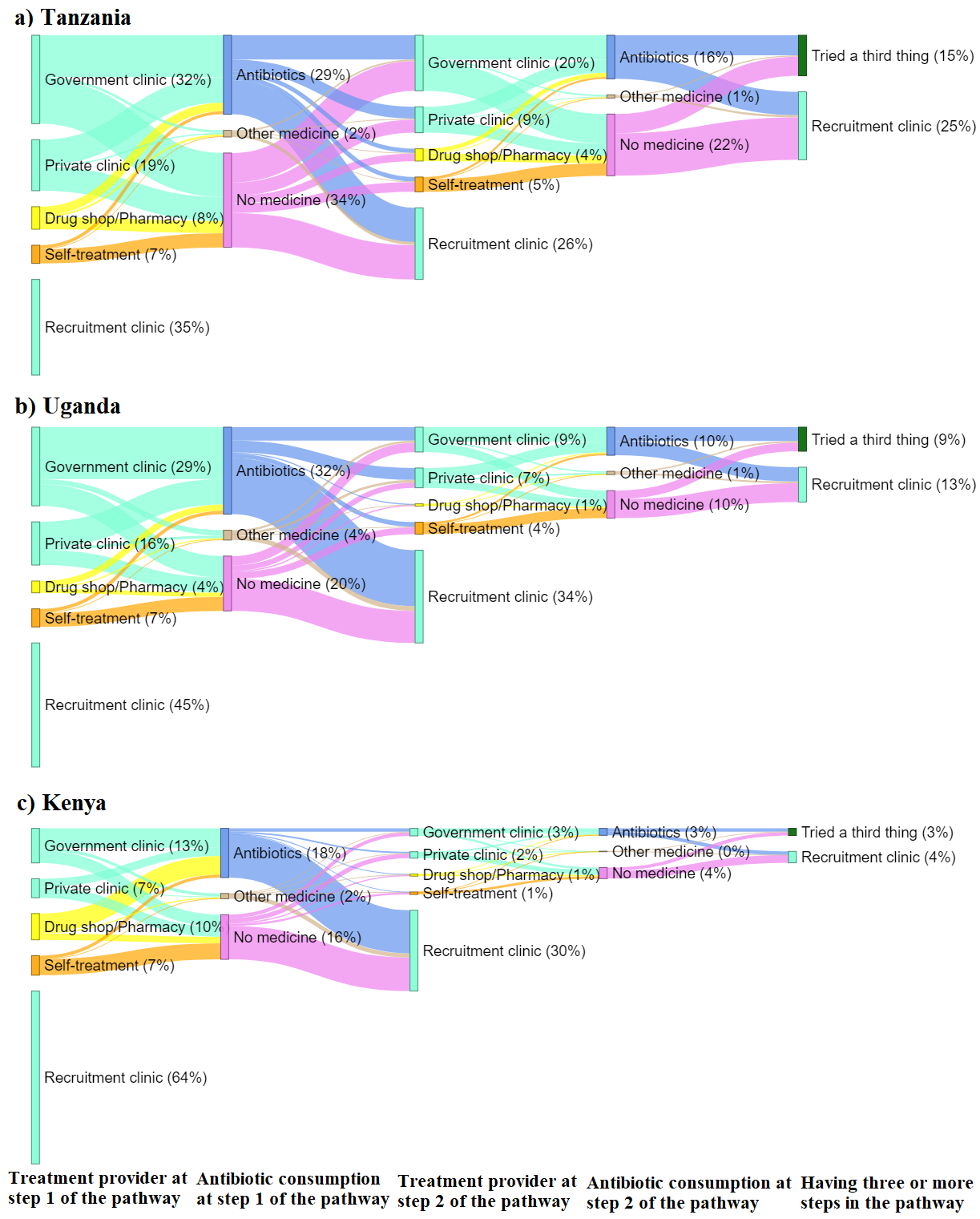
**

Figure S8. Description of the reasons for choosing the type of treatment in step 1 of the pathway by country


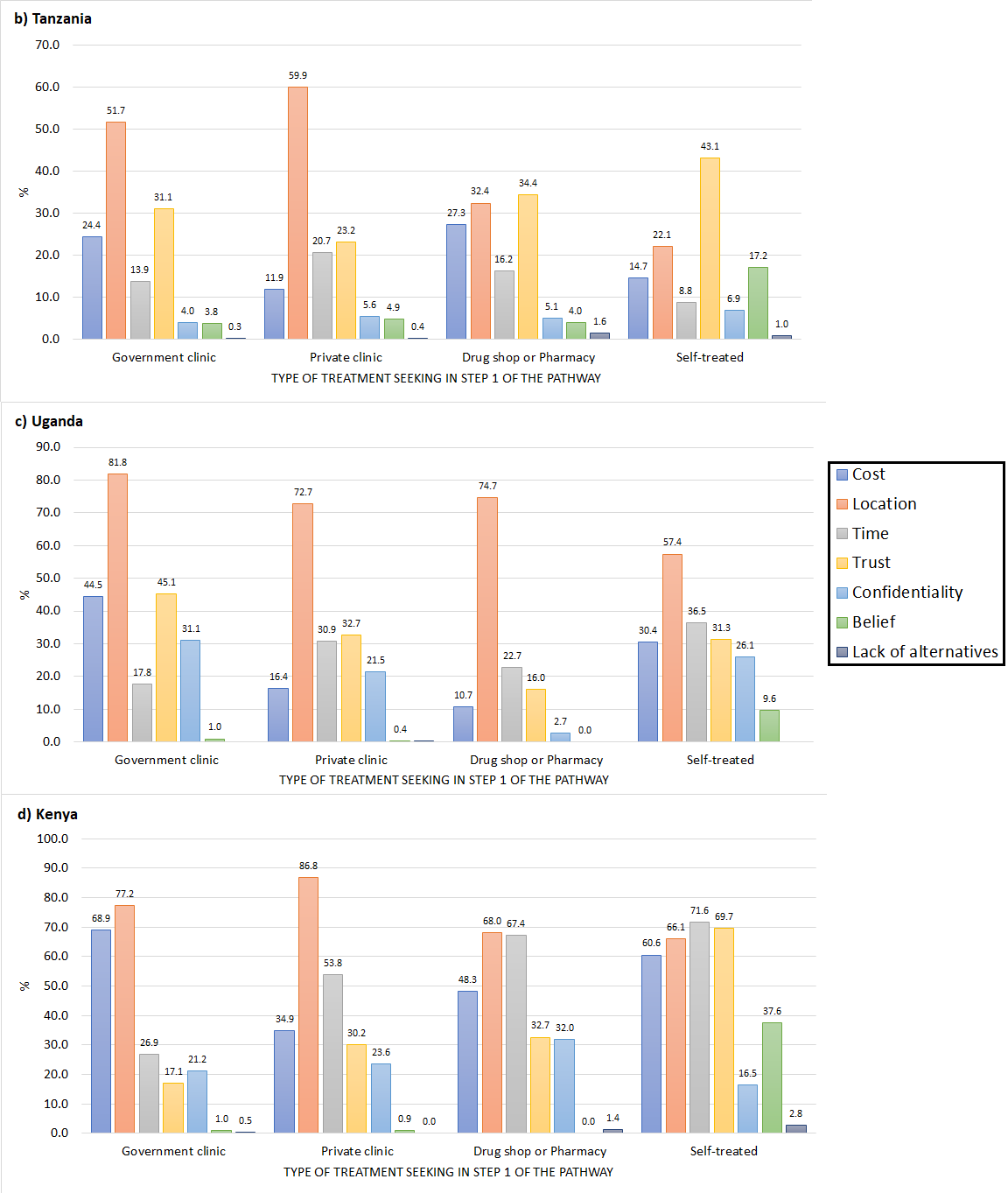


**Figure S9: Coefficients (ORs) from multivariable regression models predicting self-treatment in the first step of the pathway (N=6,608)**


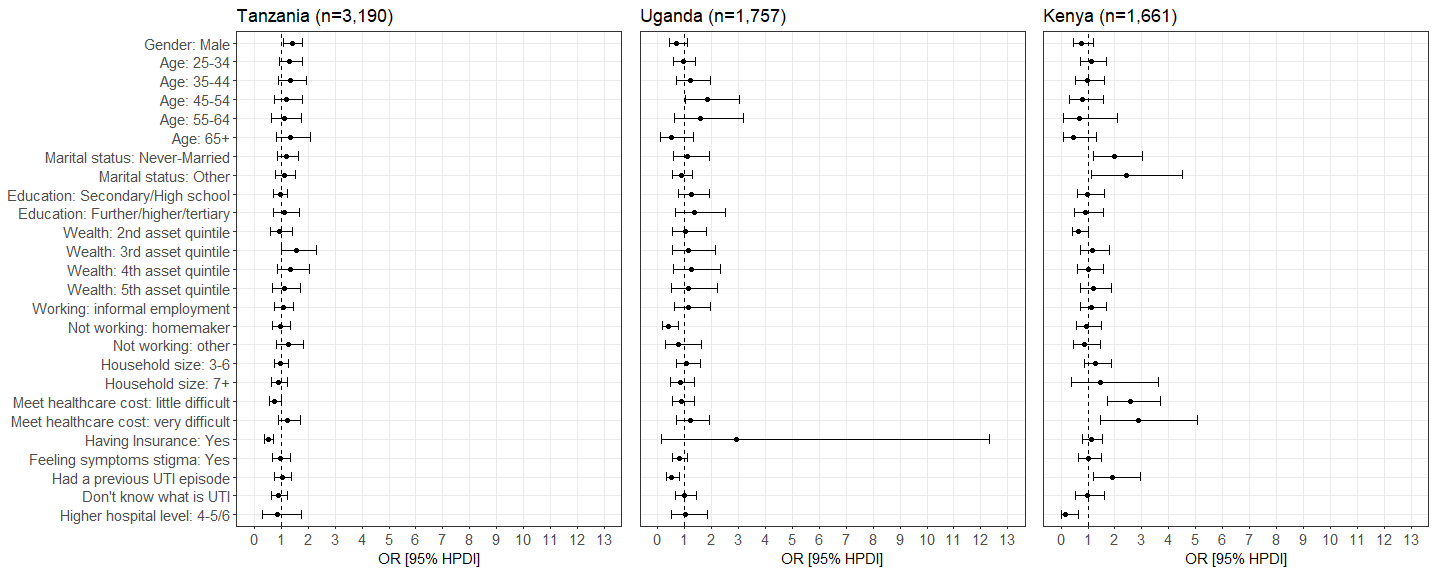
Dashed line indicates 95% Highest Posterior Density interval (HPDI) crossing 1.

**Figure S10.Coefficients (ORs) from multivariable regression models predicting having two or more steps in the pathway** **(N=6,608)**


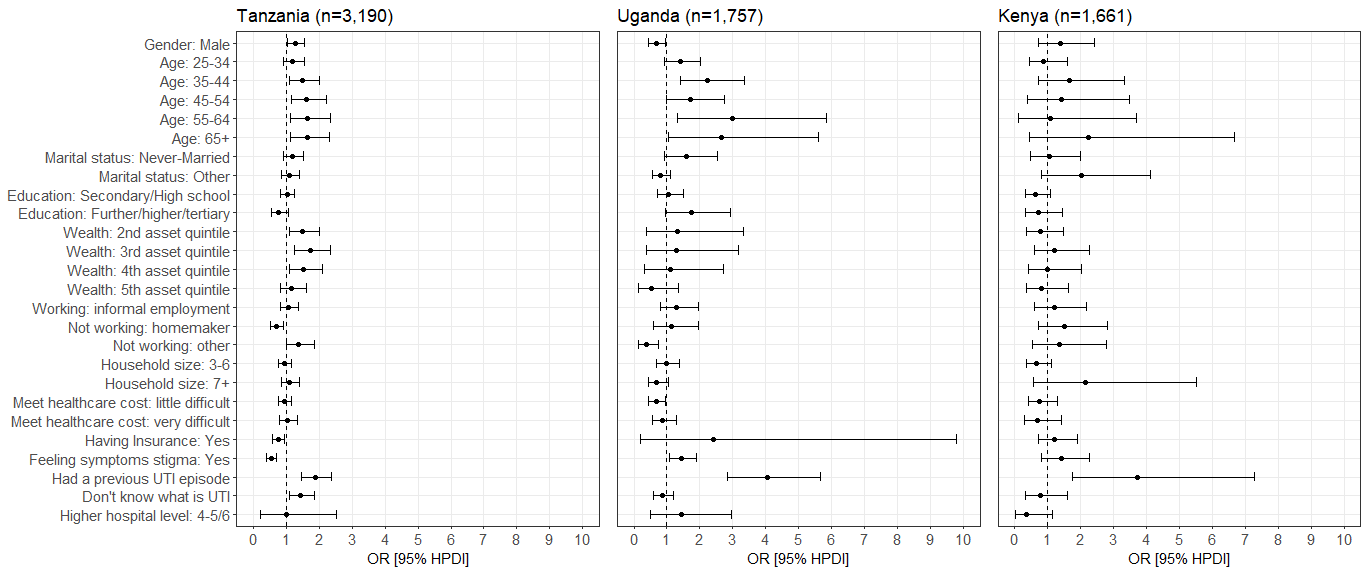
Dashed line indicates 95% Highest Posterior Density interval (HPDI) crossing 1.

**Figure S11.Coefficients (ORs) from multivariable regression models predicting antibiotic consumption in step 1 or 2 of the pathway (N=3,546)**

**
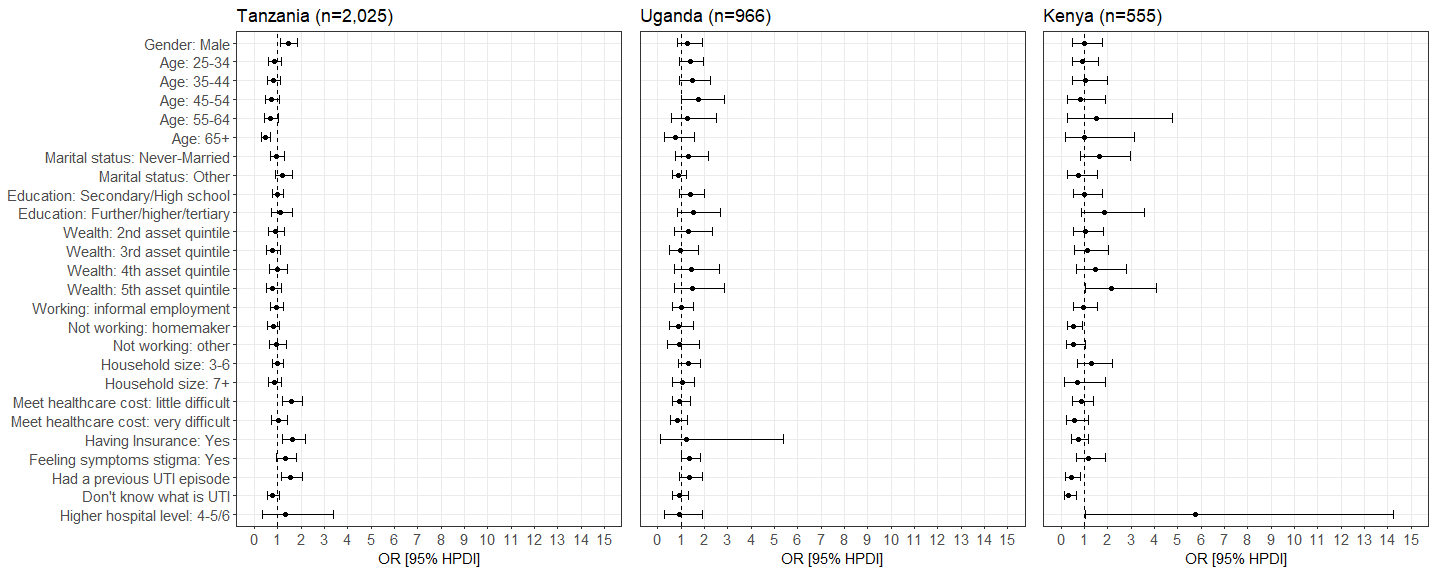
**

Dashed line indicates 95% Highest Posterior Density interval (HPDI) crossing 1

**Figure S12: Sankey plot describing patient treatment seeking pathways for UTI-like symptoms for those who attended clinics at step 1 (N=1,127)**

**
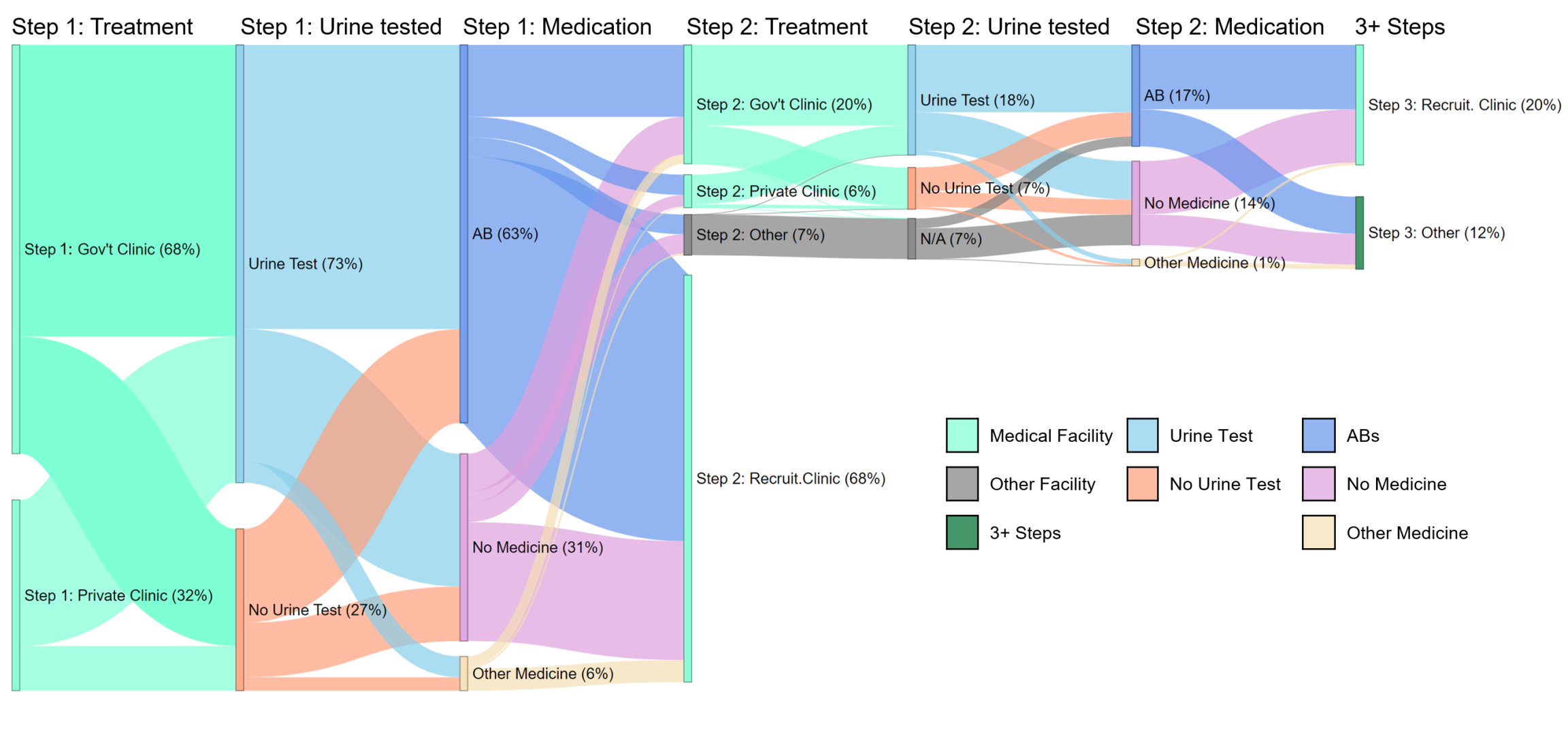
**

Figure notes: Sub-sample of patients who attended either government or private clinic as a first step and took part in phase 2 of the study (conducted Jan- Sept 2020). The date restriction is due to a new question on testing being introduced only in phase 2.

Table S5: **Medications reportedly taken by patients before coming to the recruitment clinic at step 1.**

| **Drug name**  **(brand names)** | **Freq^1^** | **Type of drug** |
| --- | --- | --- |
| **Antibiotics/**  **antimicrobials** |  |  |
| Amoxicillin | 758 | AB |
| Amoxicillin/ Clavulanic Acid (Amoxiclav) | 88 | AB |
| Ampicillin | 48 | AB |
| Ampicillin-Cloxacillin (Ampiclox) | 58 | AB |
| Azithromycin (Azuma) | 78 | AB |
| Ceftriaxone | 85 | AB |
| Cefuroxime | 2 | AB |
| Cephalexine | 19 | AB |
| Ciprofloxacin | 687 | AB |
| Cotrimoxozole (Septrine) | 40 | AB |
| Doxycycline | 143 | AB |
| Erythromycin | 98 | AB |
| Gentamicin | 27 | AB |
| Levofloxacin | 4 | AB |
| Metronidazole (Flagyl) | 248 | AB |
| Nitrofurantoin | 40 | AB |
| Tetracycline | 21 | AB |
| Tinidazole | 7 | Antimicrobial |
| **Other medicines** |  |  |
| Amlodipine | 2 | High blood pressure |
| Benzodiazepine | 13 | Central nervous system depressive drugs |
| Captopril | 1 | hypertension |
| Clotrimozole (Clotrinozole, Cotrim) | 18 | Anti-fungal |
| Diclofenac | 50 | Pain relief |
| Fluconazole | 18 | Antifungal |
| Lorsatan (Lorsa) | 1 | High blood pressure |
| Malaria | 3 | Anti-malarial |
| Nifedepine | 3 | High blood pressure |
| Omeprazole | 26 | Stomach acid regulator |
| Paracetamol | 374 | Pain relief |
| Pregabalin | 2 | Epilepsy/  Anxiety |

^1^ Frequency of reports of having taken this drug at step 1, drug either named or identified through drug card/ drug bag.

**Figure S13: Sankey plot describing patient treatment seeking pathways for those with microbiologically confirmed UTI**

**
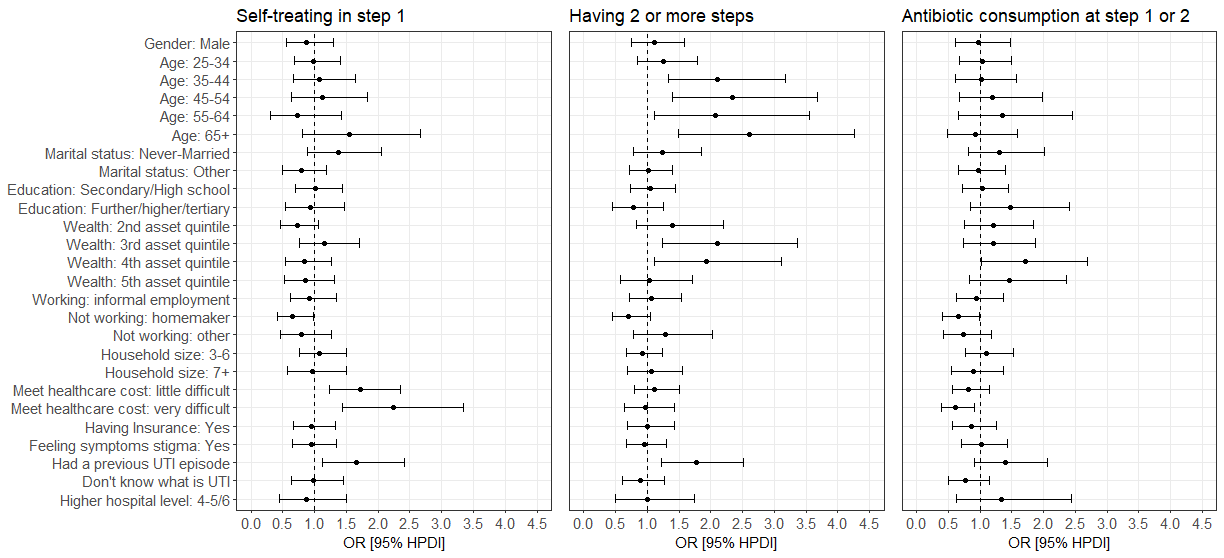
**

**References**

1. Howe LD, Galobardes B, Matijasevich A, Gordon D, Johnston D, Onwujekwe O, et al. Measuring socio-economic position for epidemiological studies in low- and middle-income countries: a methods of measurement in epidemiology paper. International Journal of Epidemiology. 2012;41:871–86.

2. Vyas S, Kumaranayake L. Constructing socio-economic status indices: how to use principal components analysis. Health Policy and Planning. 2006;21:459–68.

3. Naveed TA, Gordon D, Ullah S, Zhang M. The Construction of an Asset Index at Household Level and Measurement of Economic Disparities in Punjab (Pakistan) by using MICS-Micro Data. Social Indicators Research. 2021;155:73–95.

4. Lund C, Cois A. Simultaneous social causation and social drift: Longitudinal analysis of depression and poverty in South Africa. Journal of Affective Disorders. 2018;229:396–402.

5. Williams B, Onsman A, Brown T. Exploratory factor analysis: A five-step guide for novices. Australasian Journal of Paramedicine. 2010;8:1–13.

6. Chan LL, Idris N. Validity and Reliability of The Instrument Using Exploratory Factor Analysis and Cronbach’s alpha. International Journal of Academic Research in Business and Social Sciences. 2017;7:400–10.

7. Valpine P de, Turek D, Paciorek CJ, Anderson-Bergman C, Lang DT, Bodik R. Programming With Models: Writing Statistical Algorithms for General Model Structures With NIMBLE. http://dx.doi.org/101080/1061860020161172487. 2017;26:403–13.
